# Clinical effects of N-acetylcysteine in ICU patients with respiratory disease: a systematic review and meta-analysis

**DOI:** 10.36416/1806-3756/e20250438

**Published:** 2026-03-05

**Authors:** Mehrnaz Amiri, Mahdis Cheraghi, Farima Khalili, Lia D’Ambrosio, Rosella Centis, Anh Tuan Dinh-Xuan, Vishwanath Venketaraman, Mohammad Javad Nasiri, Giovanni Battista Migliori

**Affiliations:** 1. Department of Microbiology, School of Medicine, Shahid Beheshti University of Medical Sciences, Tehran, Iran.; 2. Public Health Consulting Group, Lugano, Switzerland.; 3. Servizio di Epidemiologia Clinica delle Malattie Respiratorie, Istituti Clinici Scientifici Maugeri - IRCCS - Tradate, Italia.; 4. Physiology Department - Functional Explorations, Cochin Hospital, GHU AP-HP Centre, Paris Cité University, Paris, France.; 5. College of Osteopathic Medicine of the Pacific, Western University of Health Sciences, Pomona (CA) USA.

**Keywords:** Acetylcysteine, Mucolytic agents, Intensive care units, Clinical study, Hospital mortality

## Abstract

**Objective:**

Critically ill patients with respiratory disease often experience impaired airway clearance, which contributes to adverse clinical outcomes. N-acetylcysteine (NAC) has mucolytic and antioxidant properties, and may have therapeutic potential in this setting. This study evaluated the impact of NAC on clinical outcomes in ICU patients with underlying respiratory disease.

**Methods:**

A systematic literature search was conducted in PubMed/MEDLINE, Embase, the Cochrane Central Register of Controlled Trials, Scopus, and Web of Science from January of 2000 to July of 2025 in order to identify randomized controlled trials evaluating NAC in ICU patients with respiratory conditions. Primary outcomes included hospital mortality, duration of mechanical ventilation, ICU length of stay (LOS), and hospital LOS. Data were synthesized using fixed- or random-effects models based on heterogeneity. Subgroup analyses were performed based on the route of NAC administration.

**Results:**

Five randomized controlled trials comprising 340 patients were included. Hospital mortality was 44.76% in the NAC group and 47.61% in the control group (OR = 0.87; 95% CI: 0.49-1.53). No significant differences were observed in ventilation duration (mean difference [MD] = 0.79 days; 95% CI: −2.87 to 4.44) or ICU LOS (MD = 0.21 days; 95% CI: −3.75 to 4.17). However, NAC was associated with a shorter hospital stay (MD = −3.84 days; 95% CI: −7.44 to −0.24). Subgroup analysis suggested variability in mortality outcomes based on administration route.

**Conclusions:**

NAC may reduce hospital LOS in critically ill patients with respiratory disease, although its effects on mortality and ventilation duration remain inconclusive. These findings may inform future research, particularly in patients with post-infectious lung damage such as that seen in COVID-19 or tuberculosis.

## INTRODUCTION

Critically ill patients with respiratory disease often experience impaired mucociliary clearance, mucus plugging, and ventilator-associated pneumonia, all of which contribute to prolonged mechanical ventilation, longer ICU stays, and higher mortality.^1^ ^-^ ^4^ Those with prior COVID-19 or pulmonary tuberculosis are especially vulnerable because of lasting lung damage-such as fibrosis and chronic sputum production-which can lead to recurrent ICU admissions even after apparent recovery.^5^ ^-^ ^12^

N-acetylcysteine (NAC) is a widely used mucolytic and antioxidant agent that is thought to improve airway clearance and mitigate inflammation in respiratory disease.^13^ ^,^ ^14^ Not only does NAC disrupt disulfide bonds in mucus to reduce viscosity but also exhibits antioxidant and anti-inflammatory effects that may benefit patients with chronic lung inflammation, including those recovering from COVID-19 or tuberculosis-related damage.^15^ ^-^ ^24^

Despite its frequent use, the effect of NAC on clinical outcomes in critically ill patients remains uncertain. Existing studies vary in patient populations, dosing strategies, and reported endpoints, and no previous meta-analysis has comprehensively evaluated the outcomes of NAC therapy in ICU patients with underlying respiratory disease.^18^ ^,^ ^19^

This systematic review and meta-analysis sought to evaluate the effects of NAC on hospital mortality and duration of mechanical ventilation, as well as on hospital and ICU length of stay (LOS) in critically ill patients with respiratory disease.

## METHODS

### Search strategy

We conducted a comprehensive literature search across five major databases-PubMed/MEDLINE, Embase, the Cochrane Central Register of Controlled Trials, Scopus, and Web of Science-from January 1, 2000 to July 1, 2025 to identify randomized controlled trials (RCTs) evaluating the clinical effects of NAC in ICU patients with underlying respiratory disease. 

The following search terms were searched in each database separately to assess the efficacy of NAC: “Mucolytic,” “Acetylcysteine,” “NAC,” “Outcome,” “Prognosis,” “Clinical outcome,” “Treatment outcome,” “Disease outcome,” “Patient outcome,” “Recovery,” “Response to treatment,” “Death,” “Mortality,” “Favorable,” “Unfavorable,” “Failure,” “Critically ill,” “Intensive care,” “Critical illness,” “Critically ill patients,” “ICU,” “Intensive care unit,” “Mechanical ventilation,” “Multiorgan failure.” 

This study was conducted and reported by the Preferred Reporting Items for Systematic Reviews and Meta-Analyses statement^25^ and was registered with the International Prospective Register of Systematic Reviews (PROSPERO ID: CRD420251055911). 

### Study selection

**Figure 1:**
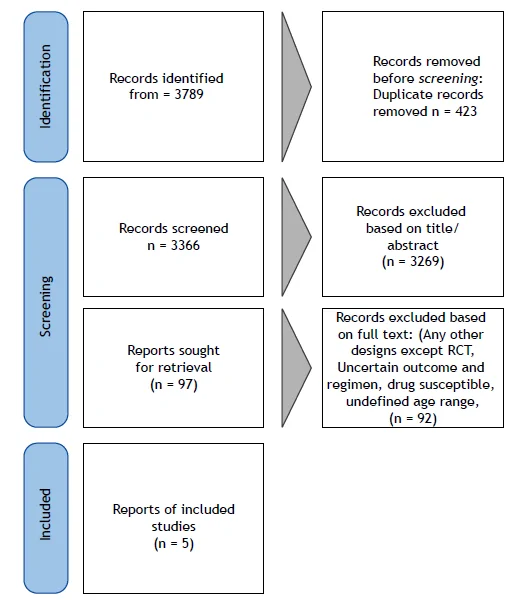
Flow chart of study selection. RCTs: randomized controlled trials.

The 3,789 records were consolidated, and duplicates were eliminated using EndNote X8 (Thomson Reuters, Toronto, ON, Canada). Two reviewers independently screened the titles and abstracts of the remaining articles. Any disagreements were resolved by a third reviewer. The full text of the remaining records was screened and assessed. All potentially eligible studies with any remaining discrepancies were resolved through a third reviewer (Figure 1). 

Eligible studies were selected on the basis of the Population, Intervention, Comparator, and Outcome framework:

- study design-RCTs assessing the clinical outcomes of NAC use in critically ill patients admitted to the ICU.
- patients-Individuals > 14 years of age with underlying respiratory diseases leading to ICU admission.
- interventions-NAC administered as a mucolytic agent, in addition to standard care.
- comparisons-Placebo administered alongside standard care or the treatment regimens without NAC.
- outcomes-Primary outcomes included hospital mortality, duration of mechanical ventilation, ICU LOS, and hospital LOS.

Exclusion criteria were as follows: other types of studies rather than RCTs (cohort studies, case-control studies, cross-sectional studies, case reports/series, reviews, editorials, conference abstracts, etc.); studies focusing exclusively on pediatric populations; and studies involving patients who were not critically ill. Studies that failed to report on hospital mortality, duration of mechanical ventilation, ICU LOS, or hospital LOS as treatment outcomes were also excluded. 

### Data extraction

Two of the authors systematically extracted data into a predefined spreadsheet using Microsoft Excel (Microsoft, Redmond, WA, USA). Disagreements were resolved by consultation with a third reviewer. The spreadsheet included the following: name of the first author; publication year; study design; study period; country and setting; patient demographics (age, sex, BMI, underlying condition, APACHE II score, SOFA score, and baseline PaO_2_/FiO_2_); treatment characteristics (number of cases and controls; dose; frequency; route of administration; duration of intervention; and co-interventions); and clinical outcomes (hospital mortality, ICU mortality, hospital LOS, and ICU LOS). 

### Quality assessment

The quality of the included studies was assessed by two reviewers using the Cochrane Risk of Bias tool, with any discrepancies being resolved by a third reviewer. This tool evaluates key domains, including random sequence generation, allocation concealment, blinding of participants and personnel, blinding of outcome assessors, completeness of outcome data, selective reporting, and other potential sources of bias. Each study was classified by risk of bias, as follows: low risk (no major concerns), high risk (significant concerns), or unclear risk (insufficient information). 

### Data analysis

Statistical analyses were performed with Comprehensive Meta-Analysis software, version 3.0 (Biostat Inc., Englewood, NJ, USA). We calculated mean differences (MDs), ORs, and 95% CIs for the effect estimates. The choice between a random-effects or fixed-effects model was based on heterogeneity, which was assessed by Cochran’s Q test and the I^2^ statistic. Publication bias was evaluated by Begg’s test, with a value of p < 0.05 being considered significant. 

## RESULTS

Figure 1 illustrates the systematic study selection process. An initial search yielded 3,789 records, of which 423 duplicates were removed. After screening the titles and abstracts of the remaining 3,366 articles, 97 full-text articles were assessed for eligibility. Following full-text review, 5 studies met the inclusion criteria and were therefore included in the final analysis.^26^ ^-^ ^29^ ^,^ ^37^

### Study characteristics

Table 1 summarizes the main characteristics of the included studies: 5 RCTs with a total of 340 patients, including 222 males. The studies were published between 2015 and 2023, and were conducted in two countries. The number of participants per study ranged from 40 to 140,^26^ ^,^ ^27^ with a mean age of 54.3 years (range, 45.2-61.8 years). 

**Table 1:** Characteristics of included studies.

Authors	Study Design	Country	Study period	Number of patients (case/control)	Mean age	No. of male patients	Underlying condition	APACHE II score	SOFA score	Baseline PaO_2_/FiO_2_	Prophylaxis/Treatment	Type of Mucolytic	Dose and Frequency	Route of administration	Duration of Treatment
Masoompour et al.^26^	RCT	Iran	January of 2012 to June of 2012	40 (20/20)	55.15	19	hospitalized in the ICU (pneumonia, poisoning [carbon monoxide and drugs], and sepsis)	NM	NM	NM	T	NAC	2 mL of NAC 20% with 8 mL normal saline TDS	nebulizer	1 day
Sharafkhah et al.^37^	RCT	Iran	March of 2014 to June of 2016	60 (30/30)	49.43 ± 18.7	43	At risk of VAP*	21.7 ± 7.5 (NAC), 22.7 ± 8.0 (control)	NM	NM	P	NAC	600 mg BD	p.o.	during the period of invasive mechanical ventilation
Ghorbi et al. ^29^	RCT	Iran	2020	60 (30/30)	45.29 ± 15.91	32	hospitalized in the ICU (ARDS)	13.63 ± 2.42 (NAC), 14.16 ± 0.79 (control)	NM	123.66 ± 7.39 (NAC), 123.8 ± 7.04 (control)	T	NAC	150 mg/kg + 50 mg/kg	i.v.	150 mg/kg on day one and 50 mg/kg for 3 days
Gamarra-Morales et al. ^28^	RCT	Spain	March of 2020 to June of 2020	140 (72/68)	61.8	106	hospitalized in the ICU with COVID-19	13.5 (NAC), 17.5 (control)	4.51 (NAC), 5.01 (control)	168.6 (NAC), 179 (control)	T	NAC	loading: 150 mg/kg + 50 mg/kg- maintenance: 50 mg/kg	i.v.	15 min + 4 h + 72 h
Rahimi et al. ^27^	RCT	Iran	2020	40 (20/20)	58.52 ± 15.73	22	hospitalized in the ICU with a positive COVID-19 test	NM	NM	NM	T	NAC	300 mg/kg	i.v.	1 day

RCT: randomized controlled trial; NM: not mentioned; T: treatment; BD: twice daily; NAC: n-acetylcysteine; TDS: three times a day; P: prophylaxis; and VAP: ventilator-associated-pneumonia. *Reasons for ICU admission included trauma, respiratory failure, and neurological (in most of the patients), heart, gastrointestinal, and endocrine conditions, as well as infection.

Among patients admitted to the ICU, nearly all had an underlying respiratory condition or were hospitalized with newly diagnosed respiratory disease. In 3 studies, all patients were diagnosed with either COVID-19 (n = 180)^27^ ^,^ ^28^ or ARDS (n = 60).^29^ The predominant diagnoses in the other studies included pneumonia, respiratory failure, chest trauma, and infection. Masoompour et al.^26^ noted that there were no significant differences in outcomes based on underlying diagnosis. In most of the studies, the control group received placebo in addition to the standard regimen. Notably, the route of administration, dose, and frequency of NAC were consistent between intervention and control arms within each study. 

*Quality assessment*

The quality of the included studies was assessed with the Cochrane Risk of Bias tool (Table 2). Of the 5 studies included in the present systematic review and meta-analysis, 4 were judged to have a low risk of bias across all domains. One study,^28^ however, was assessed as having a high risk of bias in allocation concealment, blinding of participants and personnel, and blinding of outcome assessment. 

**Table 2:** Quality of included studies, as assessed by the Cochrane Risk of Bias tool.

Authors	Random Sequence Generation	Allocation Concealment	Blinding of Participants	Blinding of Outcome Assessors	Incomplete Outcome Data	Selective Reporting	Other bias
Masoompour et al.^26^	Low	Low	Low	Low	Low	Low	Low
Sharafkhah et al.^37^	Low	Low	Low	Low	Low	Low	Low
Ghorbi et al.^29^	Low	Low	Low	Low	Low	Low	Low
Gamarra-Morales et al.^28^	Low	High	High	High	Low	Low	Low
Rahimi et al.^27^	Low	Low	Low	Low	Low	Low	Low

### Evaluating the uses of NAC in ICU patients

The clinical efficacy of NAC was evaluated across four key outcomes: hospital mortality, duration of mechanical ventilation, ICU LOS, and hospital LOS. 

**Figure 2:**
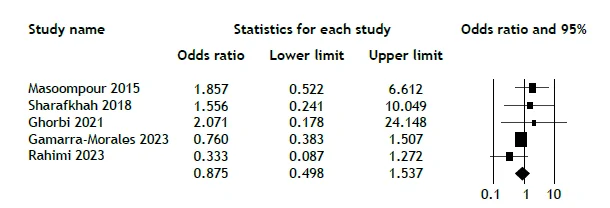
Comparison of hospital mortality between the N-acetylcysteine and control groups in the included studies.

All 5 included studies reported on hospital mortality, with pooled analysis showing a mortality rate of 44.76% in the NAC group and of 47.61% in the control group. This difference was not significant (OR = 0.87; 95% CI: 0.49-1.53; Figure 2). 

Data from 3 studies assessing the duration of mechanical ventilation showed no significant difference between groups (MD = 0.79 days; 95% CI: −2.87 to 4.44; Table 3). Similarly, analysis of 4 studies reporting on ICU LOS revealed no significant reduction associated with NAC treatment (MD = 0.21 days; 95% CI: −3.75 to 4.17; Table 3). However, pooled data from 2 studies assessing hospital LOS indicated a significantly shorter LOS in the NAC group than in the control group (MD = −3.84 days; 95% CI: −7.44 to −0.24; Table 3). 

**Table 3:** Different outcomes of included studies.

Outcome	No. of participants	No. of studies	Effect Size % (95% Cl)
Ventilation duration	260	3	MD: 0.789 [−2.87 to 4.44]
ICU LOS	300	4	MD :0.21 [−3.75 to 4.17]
Hospital LOS	100	2	MD: −3.84 [−7.44 to −0.24]

MD: mean difference; and LOS: length of stay.

### Subgroup analysis by route of administration

A subgroup analysis explored whether the route of NAC administration influenced hospital mortality. Of the 5 included studies, 3 utilized intravenous administration, whereas 2 employed non-intravenous routes (oral or nebulized). The pooled mortality estimate was OR = 0.637 (95% CI: 0.27-0.88) for intravenous NAC and OR = 0.257 (95% CI: 0.03-0.74) for non-intravenous routes. These findings suggest potential variability in treatment effect by administration route; however, the results should be interpreted cautiously because of potential confounding by baseline disease severity and the absence of formal interaction testing. 

## DISCUSSION

This systematic review and meta-analysis evaluated the clinical efficacy of NAC in critically ill patients with underlying respiratory disease admitted to the ICU. Our findings suggest that while NAC does not significantly reduce hospital mortality, duration of mechanical ventilation, or ICU LOS, it may be associated with a modest reduction in hospital LOS. However, the relatively small number of studies may have limited its statistical power. This issue will be further discussed as a limitation. 

The role of NAC in the ICU remains relatively understudied, despite its widespread use in chronic respiratory diseases such as COPD, bronchiectasis, and cystic fibrosis. Our analysis helps address this gap by focusing on a population at an elevated risk of mucus-related complications-critically ill individuals requiring intensive support. Although the observed trend toward reduced hospital LOS may indicate potential benefit, these findings should be interpreted cautiously, given that no studies assessed direct mucolytic outcomes such as mucus viscosity, volume, or clearance. Therefore, the mechanism of action remains speculative in this context. 

Subgroup analysis suggested that intravenous administration of NAC was associated with higher mortality than were non-intravenous routes. However, this result is likely influenced by confounding by indication, with more severely ill patients preferentially receiving intravenous therapy. Differences in baseline disease severity, treatment protocols, and study-level heterogeneity further limit the interpretability of this observation. No formal test for interaction was performed, and these exploratory findings require validation in stratified analyses. 

Although our findings may have theoretical relevance for patients with structural lung damage caused by COVID-19 or tuberculosis, this application remains speculative. Both conditions are known to result in fibrosis, bronchiectasis, and impaired mucociliary clearance.^30^ ^-^ ^34^ Even after clinical or microbiological recovery, residual pulmonary dysfunction can persist, leading to chronic sputum production and respiratory compromise. In such contexts, the mucolytic and antioxidant properties of NAC may have therapeutic potential, but none of the included studies specifically enrolled patients with post-COVID-19 or post-tuberculosis lung disease, and no subgroup analyses were based on etiology. As such, extrapolation to these populations should be avoided without direct evidence. Further targeted research is needed to confirm any benefit of NAC in these settings.^35^ ^,^ ^36^

The present study has several limitations. Although no significant differences were observed in some of the analyzed outcomes, the relatively small number of studies and participants included in our meta-analysis may have limited its statistical power. Therefore, the absence of statistical significance should be interpreted with caution and does not necessarily indicate a lack of true effect, but rather insufficient cumulative evidence. Considerable heterogeneity in patient populations and baseline diagnoses limits the generalizability of the findings. Notably, none of the included studies reported tuberculosis status or specifically enrolled patients with post-tuberculosis lung disease, precluding disease-specific conclusions. Furthermore, variability in NAC dosing, treatment duration, and routes of administration introduces potential confounding, and no direct assessments of mucolytic efficacy were reported, such as mucus volume, viscosity, or airway clearance. 

NAC may confer modest benefits in reducing hospital LOS among critically ill patients with respiratory disease, although its effects on mortality and mechanical ventilation duration appear limited. These findings may be of interest in the management of patients with post-COVID-19 or post-tuberculosis lung sequelae, in whom mucus hypersecretion and oxidative stress contribute to prolonged recovery. However, given the limited data and indirect nature of the outcomes, the role of NAC as an adjunct therapy in this context remains hypothetical and warrants further investigation in larger, well-designed RCTs. 
